# Exercise Training Attenuates Obesity-Induced Skeletal Muscle Remodeling and Mitochondria-Mediated Apoptosis in the Skeletal Muscle

**DOI:** 10.3390/ijerph15102301

**Published:** 2018-10-19

**Authors:** Jun-Won Heo, Su-Zi Yoo, Mi-Hyun No, Dong-Ho Park, Ju-Hee Kang, Tae-Woon Kim, Chang-Ju Kim, Dae-Yun Seo, Jin Han, Jin-Hwan Yoon, Su-Jeen Jung, Hyo-Bum Kwak

**Affiliations:** 1 Department of Kinesiology, Inha University, Incheon 22212, Korea; gjwnsdnjs03@naver.com (J.-W.H.); susie_737@naver.com (S.-Z.Y.); 77nodaji@hanmail.net (M.-H.N.); dparkosu@inha.ac.kr (D.-H.P.); 2 Department of Pharmacology and Medicinal Toxicology Research Center, Inha University School of Medicine, Incheon 22212, Korea; johykang@inha.ac.kr; 3 Department of Physiology, College of Medicine, Kyung Hee University, Seoul 02447, Korea; twkim0806@naver.com (T.-W.K.); changju@khu.ac.kr (C.-J.K.); 4 National Research Laboratory for Mitochondrial Signaling, Department of Physiology, College of Medicine, Cardiovascular and Metabolic Disease Center, Inje University, Busan 47392, Korea; sdy925@gmail.com (D.-Y.S.); phyhanj@gmail.com (J.H.); 5 Department of Kinesiology, Hannam University, Daejeon 34430, Korea; yoonjh@hannam.ac.kr; 6 Department of Leisure Sports, Seoil University, Seoul 02192, Korea; sujeenj@nate.com

**Keywords:** obesity, exercise, skeletal muscle, mitochondria, apoptosis

## Abstract

Obesity is characterized by the induction of skeletal muscle remodeling and mitochondria-mediated apoptosis. Exercise has been reported as a positive regulator of skeletal muscle remodeling and apoptosis. However, the effects of exercise on skeletal muscle remodeling and mitochondria-mediated apoptosis in obese skeletal muscles have not been clearly elucidated. Four-week-old C57BL/6 mice were randomly assigned into four groups: control (CON), control plus exercise (CON + EX), high-fat diet (HFD), and HFD plus exercise groups (HFD + EX). After obesity was induced by 20 weeks of 60% HFD feeding, treadmill exercise was performed for 12 weeks. Exercise ameliorated the obesity-induced increase in extramyocyte space and a decrease in the cross-sectional area of the skeletal muscle. In addition, it protected against increases in mitochondria-mediated apoptosis in obese skeletal muscles. These results suggest that exercise as a protective intervention plays an important role in regulating skeletal muscle structure and apoptosis in obese skeletal muscles.

## 1. Introduction

Obesity is rapidly emerging as one of the most important health problems of the 21st century. Overweight and obesity, which have affected approximately 1.3 billion persons (approximately 33% of the world’s adult population) in 2005, are expected to affect approximately 3.3 billion persons (approximately 58% of the world’s adult population) by 2030 [1]. Obesity results from diverse causes, including genetic factors, environmental factors, stress, and high-fat diet (HFD), and are associated with cardiovascular diseases, type 2 diabetes, insulin resistance, sleep apnea, and cancer [2]. In the human body, the skeletal muscle is the largest organ, accounting for approximately 40–50% of the total body mass. Previous studies reported that obesity is associated with skeletal muscle remodeling [3], resulting in skeletal muscle dysfunction including abnormal protein turnover, reduced glucose uptake, decreased lipid metabolism, and impaired mitochondrial function [4,5,6].

Apoptotic signaling primarily induces apoptosis via three complex pathways: (a) cytokine/Fas receptor-driven pathway, (b) mitochondria-mediated pathway, and (c) endo(sarco)plasmic reticulum/Ca^2+^-driven pathway [7]. Among these three pathways, the mitochondria-mediated pathway, which involves the Bcl-2 family proteins, is considered the most important in controlling apoptosis in various skeletal muscle-related diseases [8,9,10,11,12,13]. The Bcl-2 pathway includes membrane-bound proteins including (a) anti-apoptotic proteins called “gatekeepers”, such as Bcl-2 and Bcl-X_L_; and (b) pro-apoptotic proteins called as “gatecrashers”, such as Bax, Bid, and Bad [7]. The ratio of pro-apoptotic to anti-apoptotic proteins controls myonuclei integrity and cellular survival by regulating mitochondrial membrane permeability [7]. Unstable mitochondrial membrane integrity, which is known as mitochondrial permeability transition pore (mPTP) opening sensitivity, induces cytochrome c release from the mitochondrial intermembrane to the cytosol and apoptosome formation and subsequently activates caspase-9 and caspase-3 [14], which results in cell death induction.

Physiologically, apoptosis plays an important role in regulating development, growth, and homeostasis and inhibiting tumorigenesis in mitotic tissues [15,16]. However, pathologically, excessive apoptosis has a negative role in maintaining the skeletal muscle function [17]. According to previous studies, there is evidence that mitochondria-mediated apoptosis is promoted further by aging [8,9,10] and skeletal muscle related-diseases, such as Duchenne and facioscapulohumeral muscular dystrophies (DMD and FSHMD) [11], muscle wasting [12], and sarcopenia [13]. However, the relationship between obesity and mitochondria-mediated apoptosis in the skeletal muscle remains unclear. Previous studies have reported conflicting results regarding the relationship between obesity and apoptosis. Some studies reported that up-regulation of pro-apoptotic signaling was induced by HFD-induced obesity [18,19]. Conversely, other studies indicated that obesity did not induce apoptotic signaling in the skeletal muscle [20]. 

It is well known that aerobic exercise training leads to improvements in cardiovascular function, aerobic capacity, and metabolic regulation [21,22,23]. In addition, regular exercise training is considered to be responsible for skeletal muscle hypertrophy [21] as well as the normalization of myocyte apoptosis in skeletal muscle [24,25]. Many previous studies have shown the effects of exercise on mitochondrial apoptotic signaling in aging and various diseases [8,26,27]. Despite the importance of exercise training in combating various skeletal muscle diseases (e.g., DMD, FSHMD, muscle wasting, and sarcopenia), the capability of exercise to attenuate skeletal muscle remodeling and apoptosis in obese skeletal muscles has not been evaluated. To our knowledge, only one study has reported that exercise training affects obesity-related mitochondria-mediated apoptosis. That study demonstrated that exercise training attenuated mitochondria-mediated apoptosis induced by obesity in the cardiac muscle but not in the skeletal muscle [20]. Therefore, we hypothesized that aerobic exercise training for 12 weeks would attenuate obesity-induced skeletal muscle remodeling and mitochondria-mediated apoptosis in the skeletal muscle.

## 2. Materials and Methods 

### 2.1. Experimental Design

Four-week-old C57BL/6 male mice were randomly divided into four groups (*n* = 8 per group): control (CON), CON plus exercise (CON + EX), HFD, and HFD plus exercise (HFD + EX). In the CON and HFD groups, a normal chow (carbohydrate 67.7% kcal, fat 11.5% kcal, protein 20.8% kcal, 3.90 kcal/g; D10001; Research Diets Inc., New Brunswick, NJ, USA) and an HFD (carbohydrate 20% kcal, fat 60% kcal, protein 20% kcal, 5.21 kcal/g; D12492; Research Diets Inc.) were provided *ad libitum* for 32 weeks. After normal diet and HFD feeding for 20 weeks, only the CON + EX and HFD + EX groups performed treadmill exercise training for 12 weeks with the diet compositions maintained. After completing HFD feeding and aerobic exercise training, the skeletal muscle (red gastrocnemius) tissues were extracted from the respective groups, and skeletal muscle morphology and apoptosis were analyzed via hematoxylin and eosin staining (myocyte number, cross-sectional area (CSA), and extramyocyte space), Western immunoblotting (Bax, Bcl-2, and cytochrome c), immunohistochemistry (IHC; cleaved caspase-3), and terminal deoxynucleotidyl transferase-mediated dUTP nick-end labeling (TUNEL) staining (apoptotic marker).

### 2.2. Animal Experiment and Ethical Approval

All animal experiments conformed to the regulations stipulated by the National Institutes of Health (NIH) and the guidelines of the Korean Academy of Medical Science. This study was approved by the Kyung Hee University Institutional Animal Care and Use Committee (Seoul, Korea) (KHUASP [SE]-14-018). Two mice were housed per cage for the normal chow groups or one mouse was housed per cage for the HFD groups under controlled temperature (20 ± 2 °C) and lighting (07:00 to 19:00 h) conditions with food and water available *ad libitum*.

### 2.3. Exercise Protocol

To test the effect of exercise on apoptosis in the obese skeletal muscles, treadmill exercise training was performed in the CON + EX and HFD + EX groups for 12 weeks after 20 weeks of HFD feeding. The mice in these two exercise groups were trained on a mice treadmill at a moderate intensity (60–75% of the maximal aerobic capacity of mice [28]). The mice were acclimated to walk on the treadmill during the first 7 days. After the acclimation period, the mice were gradually conditioned to perform exercise without inclination at 10–16 m/min, for 40–50 min/day and 6 days/week for 12 weeks.

### 2.4. Tissue Preparation

To prepare the skeletal muscle tissues for detection of morphological changes, the mice were fully anesthetized with ethyl ether after which they were transcardially perfused with 50 mM phosphate-buffered saline (PBS) and then fixed with a freshly prepared solution of 4% paraformaldehyde in 100 mM phosphate buffer (pH, 7.4). The skeletal muscles were then removed, post-fixed in the same fixative overnight, and transferred into a 30% sucrose solution for cryoprotection. In addition, to prepare the skeletal muscle tissue for Western immunoblotting, the red gastrocnemius (Type IIa) fiber was rapidly extracted and immediately frozen using liquid nitrogen.

### 2.5. Hematoxylin and Eosin Staining

To determine the effects of obesity and exercise training on the morphology of the skeletal muscle, the paraffin block section-embedded soleus muscle was cut thinly (5-µm thickness) and dried at 60 °C. Hematoxylin and eosin staining was performed at room temperature (RT) to localize the myocyte nuclei, extramyocyte space, and geometry. The myocyte number, CSA, and extramyocyte space were measured on multiple sections of the soleus using the Image J analysis program (NIH, Bethesda, MD, USA).

### 2.6. Western Immunoblotting

The protein levels of apoptotic signaling (Bax, Bcl-2, and cytochrome c) were measured through Western immunoblotting. The red gastrocnemius tissues were collected and immediately frozen at −80 °C until analysis. The tissues were homogenized with lysis buffer containing 50 mM Tris-HCl (pH, 7.5), 150 mM NaCl, 10% glycerol, 1% Triton X-100, 1.5 mM MgCl_2_·6H_2_O, 1 mM EGTA, 1 mM phenylmethylsulfonyl fluoride, 1 mM Na_2_VO_4_, and 100 mM NaF and centrifuged at 14,000 rpm for 20 min. The protein concentration was measured using a Bio-Rad colorimetric protein assay kit (Bio-Rad, Hercules, CA, USA).

A 30-μg amount of protein sample was separated on sodium dodecyl sulfate-polyacrylamide gels and transferred onto a nitrocellulose membrane. After staining with ponceau S (Sigma-Aldrich, St. Louis, MO, USA) to verify equal loading and transferring of proteins to the membranes, the membranes were incubated with 5% skim milk in Tris-buffered saline containing 0.1% Tween-20 (TBS-T) and then incubated overnight at 4 °C with the following primary antibodies: β-actin, Bcl-2, Bax, and cytochrome c (Santa Cruz Biotechnology, Dallas, TX, USA). Subsequently, the membranes were incubated for 1 h with horseradish peroxidase-conjugated anti-mouse (Vector Laboratories, Inc., Burlingame, CA, USA). After three washes in TBS-T, band detection was performed using an enhanced chemiluminescence detection kit (Thermo Fisher Scientific, Santa Clara, CA, USA). Thereafter, protein expression was detected using Chemidoc (Bio-Rad, Hercules, CA, USA). To compare the relative expressions of the proteins, detected bands were quantified using the computer-assisted Image-Pro^®^ Plus analysis system (Media Cybernetics, Inc., Silver Spring, MD, USA).

### 2.7. IHC and TUNEL Staining

Cleaved caspase-3, an apoptotic signaling marker, was assessed using IHC to determine whether obesity and exercise training affect apoptotic signaling in the skeletal muscle. For IHC analysis, 5-μm paraffin-embedded soleus sections were deparaffinized, hydrated, treated with 3% hydrogen peroxide in 0.05 M Tris-HCl (0.25% Triton X-100) for 15 min at RT, and washed three times in PBS. After blocking with a 5% diluent solution (Invitrogen, Carlsbad, CA, USA) in 0.05 M Tris-HCl containing 1 mg/mL bovine serum album, 1 mM NaF, and 0.05% Triton X-100 for 1 h at RT, the sections were incubated with cleaved caspase-3 primary antibody (Cell Signaling Technology, Beverly, MA, USA) overnight at 4 °C and rinsed three times in PBS. The signal was detected using the EnVision system-horseradish peroxidase labeled polymer anti-rabbit IgG (Dako Cytomation, Glostrup, Denmark) for 30 min at RT and 3,3′-diaminobenzidine peroxidase substrates. The sections were visualized using diaminobenzidine (Dako Cytomation, Glostrup, Denmark), counterstained with hematoxylin, dehydrated, cleared, and cover-slipped with a synthetic mounting medium. The digital images were captured using an Axioplan 2 imaging microscope (Carl Zeiss, Jena, Germany). The cleaved caspase-3-positive cells were quantified in multiple sections of the soleus by the Image J analysis program.

To detect DNA fragmentation, TUNEL staining was performed using the ApopTag^®^ Peroxidase In Situ Apoptosis Detection Kit (Chemicon International, Inc., Billerica, MA, USA) according to the manufacturer’s instructions. The images (40× magnification) were captured using the Axioplan 2 imaging system and software. The TUNEL-positive myonuclei were counted in multiple sections of the soleus and indicated as a percentage of all myonuclei.

### 2.8. Statistics

Data were presented as means ± standard errors. Two-way analysis of variance (ANOVA) was performed to determine the effects of obesity and exercise. When applicable, one-way ANOVA with Tukey’s post hoc test was used to assess the mean difference among test groups. The significance level was set at 0.05. All graphs were generated using Prism 5 (GraphPad, La Jolla, CA, USA).

## 3. Results

### 3.1. Exercise Reduces Obesity-Induced Increase in Body Weight

In the present study, we found that obesity in the mice was induced by 32 weeks of HFD feeding; the body weight of mice in the HFD group significantly increased after 32 (20 + 12) weeks of HFD feeding compared with that of the CON group (33.89 ± 0.55 g vs. 60.23 ± 0.45 g, *p* < 0.05). After 20 weeks of HFD feeding, we observed the effects of 12 weeks of exercise training on the body weight of mice with induced obesity and found that the HFD + EX group had a decreased body weight compared with the HFD group (60.23 ± 0.45 g vs. 41.36 ± 0.68 g, *p* < 0.05).

### 3.2. Exercise Ameliorates Obesity-Induced Skeletal Muscle Remodeling.

**Figure 1:**
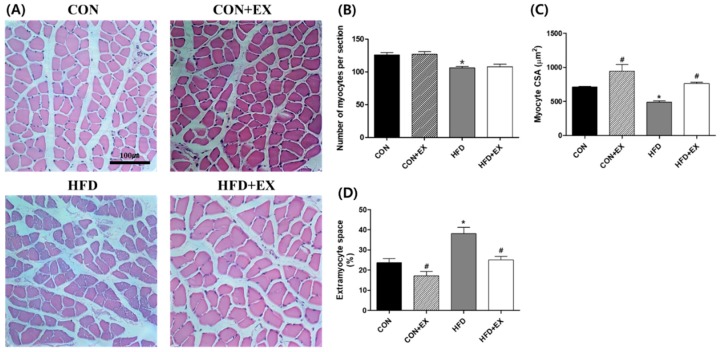
Effects of high-fat diet (HFD) feeding and exercise training on morphology (**A**), myocyte number (**B**), cross-sectional area (CSA) of myocytes (**C**), and extramyocyte space (**D**) in the skeletal muscle (hematoxylin and eosin staining). The number of myocytes (**B**) and the CSA (**C**) decreased, and the extramyocyte space (**D**) in the skeletal muscle increased in the HFD group compared with that in the control (CON) group. However, exercise training attenuated the obesity-induced skeletal muscle remodeling, demonstrating that the obesity-induced decrease in the CSA was improved, and the increase in the extramyocyte space was mitigated by exercise training in the HFD plus exercise (HFD + EX) group compared with that in the HFD group. The myocyte number, CSA, and extramyocyte space were measured in multiple sections of the soleus (four images in two mice per group were analyzed). Data are presented as means ± standard errors. * *p* < 0.05 compared with the CON group. ^#^ *p* < 0.05 compared with the matched group. Scale bar = 100 μm.

The myocyte number, CSA, and extramyocyte space were measured in the skeletal muscle (Figure 1). We found that the number of myocytes per section was lower in the HFD group than in the CON group (126.00 ± 3.45 vs. 106.00 ± 2.17, *p* < 0.05; Figure 1B). The mean myocyte CSA also decreased by 32% in the HFD group compared with that in the CON group (712.95 ± 10.50 µm^2^ vs. 490.28 ± 18.78 µm^2^, *p* < 0.05, Figure 1C). Conversely, increased extramyocyte space was confirmed in the obese myofibers compared with that in the non-obese myofibers (23.63 ± 2.09% vs. 38.16 ± 3.04%, *p* < 0.05; Figure 1D). The number of myocytes was not affected by exercise training in the HFD + EX group compared with that in the HFD group (106 ± 2.17 vs. 107.75 ± 3.82; Figure 1B). However, the myocyte CSA increased by 55% (490.28 ± 18.78 µm^2^ vs. 763.51 ± 19.12 µm^2^, *p* < 0.05; Figure 1C), and the extramyocyte space significantly decreased by 35% in the HFD + EX group compared with that in the HFD group (38.16 ± 3.04% vs. 25.06 ± 1.80%, *p* < 0.05; Figure 1D).

**Figure 2:**
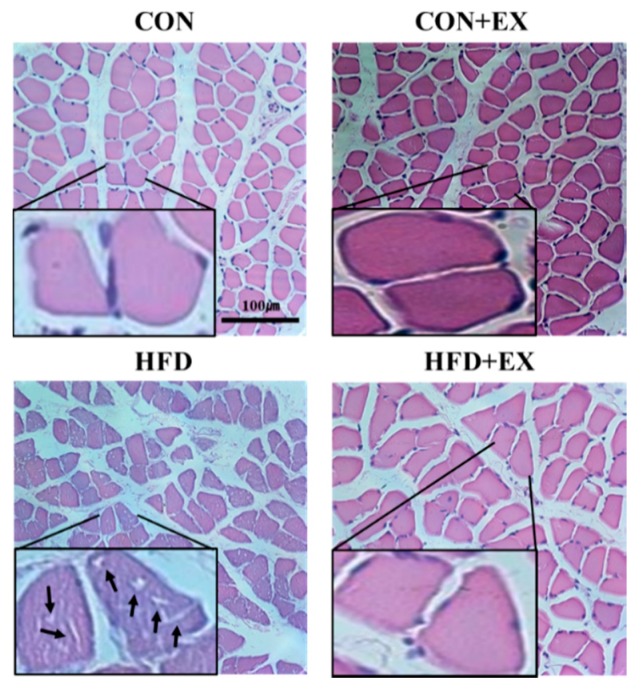
Effects of high-fat diet (HFD) feeding and exercise training on intramyocellular lipid (IMCL) infiltration in the skeletal muscle. IMCL infiltration was generated in the HFD-induced obese skeletal muscles. However, IMCL infiltration was lesser with exercise training in the HFD plus exercise (HFD + EX) group compared with that in the HFD group. Arrow indicates IMCL infiltration. IMCL infiltration was observed in sections of soleus muscle fibers. Scale bar = 100 μm.

In addition, intramyocellular lipid (IMCL) infiltration, which may be consequently associated with insulin resistance, inflammation, and skeletal muscle dysfunction [29], was observed in the obese skeletal muscles (Figure 2). However, IMCL infiltration was reduced in the HFD + EX group compared with that in the HFD group (Figure 2).

### 3.3. Exercise Attenuates Obesity-Induced Mitochondria-Mediated Apoptotic Signaling in the Skeletal Muscle

**Figure 3:**
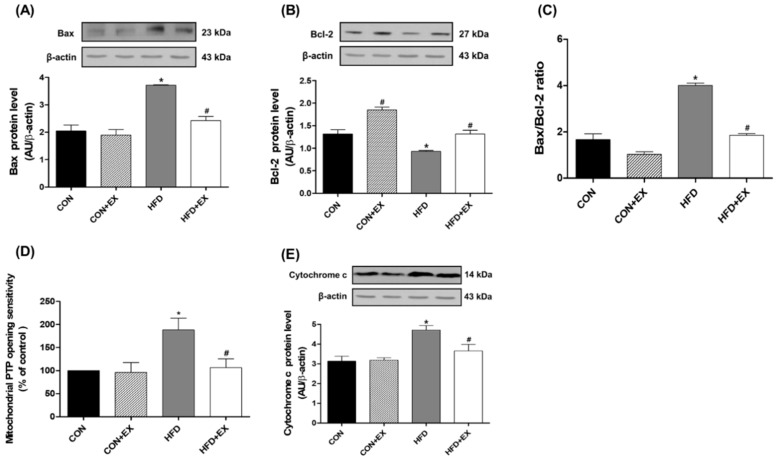
Effects of high-fat diet (HFD) and exercise training on mitochondria-mediated apoptotic signaling in the skeletal muscle (*n* = 6). HFD-induced obesity increased mitochondria-mediated apoptosis in the skeletal muscle. The Bax protein level (**A**) was increased, and the Bcl-2 protein level (**B**) was conversely decreased by obesity. The Bax/Bcl-2 ratio (**C**), mitochondrial permeability transition pore (PTP) opening sensitivity (**D**), and cytochrome c protein level (**E**) were higher in the HFD group than in the control (CON) group. However, exercise training ameliorated the obesity-induced increase in mitochondria-mediated apoptotic signaling, demonstrating that the protein levels of Bax and cytochrome c as well as the mitochondrial PTP opening sensitivity were attenuated by exercise training in the skeletal muscle. Data are presented as means ± standard errors. * *p* < 0.05 compared with the CON group. ^#^ *p* < 0.05 compared with the matched group. EX, exercise.

The Bax (pro-apoptotic protein) levels were 81% higher in the HFD group than in the CON group (*p* < 0.05, Figure 3A). In contrast, the Bcl-2 (anti-apoptotic protein) levels significantly decreased by 29% in the HFD group compared with those in the CON group (*p* < 0.05, Figure 3B), suggesting that the Bax/Bcl-2 ratio, which is used in the early stage of mitochondria-mediated apoptosis, was markedly increased by HFD-induced obesity (*p* < 0.05, Figure 3C). However, the Bax levels significantly decreased by 35% in the HFD + EX group compared with that in the HFD group (*p* < 0.05, Figure 3A). Conversely, the Bcl-2 levels were significantly greater (by 40%) in the HFD + EX group than in the HFD group (*p* < 0.05, Figure 3B), suggesting that aerobic exercise training for 12 weeks protected against obesity-induced early mitochondria-mediated apoptotic signaling.

The mPTP opening sensitivity significantly increased by 88% in the HFD group compared with that in the CON group; it dramatically decreased in the HFD + EX group compared with that in the HFD group (*p* < 0.05, Figure 3D).

In addition, the protein levels of cytochrome c, which is released by mPTP opening from the mitochondrial intermembrane to the cytosol, also presented similar results. The levels significantly increased by 50% in the HFD group compared with those in the CON group (*p* < 0.05, Figure 3E). However, the levels decreased in the HFD + EX group compared with those in the HFD group (*p* < 0.05, Figure 3E).

### 3.4. Exercise Protects against Obesity-Induced Cleaved Caspase-3 and DNA Fragmentation

**Figure 4:**
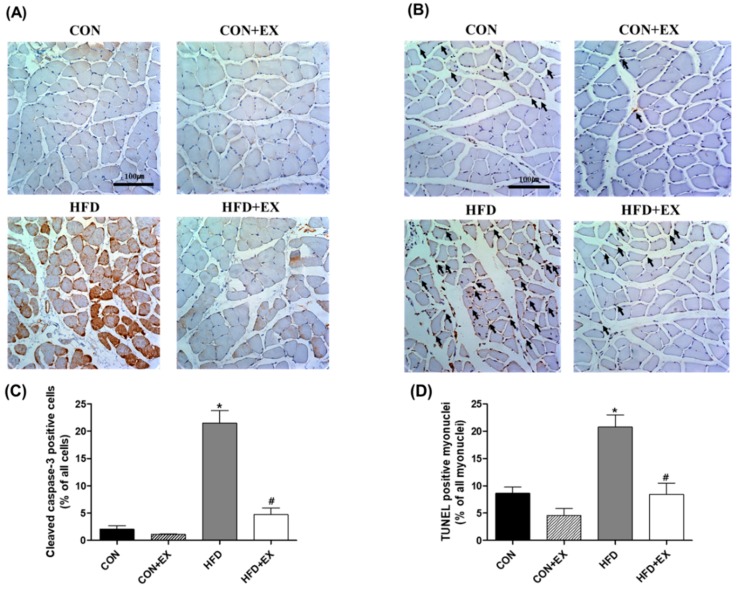
Effects of high-fat diet (HFD) and exercise training on (**A**,**C**) cleaved caspase-3 positive cells and (**B**,**D**) terminal deoxynucleotidyl transferase-mediated dUTP nick-end labeling (TUNEL)-positive myonuclei in the skeletal muscle (four images in two mice per group were analyzed). More cleaved caspase-3-positive cells in the skeletal muscle were observed in the HFD group than in the control (CON) group. In addition, the TUNEL-positive myonuclei also increased in the HFD group compared with that in the CON group. However, exercise training attenuated obesity-induced cleaved caspase-3-positive cells and TUNEL-positive myonuclei, suggesting that exercise training plays an important role in protecting against obesity-induced increase in apoptosis in the skeletal muscle. Brown color (**A**) represents cleaved caspase-3-positive myocytes. The arrow (**B**) indicates the apoptotic myonuclei. Data are presented as means ± standard errors. * *p* < 0.05 compared with the CON group. ^#^ *p* < 0.05 compared with the matched group. Ex, exercise. Scale bar = 100 μm.

We also conducted IHC for cleaved caspase-3 and TUNEL staining to measure the apoptotic cells and myonuclei in the skeletal muscle. The cleaved caspase-3 positive cells increased in the HFD group compared with those in the CON group (2.02 ± 0.67% vs. 21.48 ± 2.31%, *p* < 0.05; Figure 4A). Additionally, increased TUNEL-positive myonuclei were found in the obese skeletal muscles compared with those in the normal skeletal muscles (8.64 ± 1.11% vs. 20.81 ± 2.19%, *p* < 0.05; Figure 4B). However, the 12-week exercise training attenuated the obesity-induced increase in cleaved caspase-3 positive cells and TUNEL-positive myonuclei (21.48 ± 2.31% vs. 4.74 ± 1.21%; 20.81 ± 2.19% vs. 8.45 ± 2.04%, respectively; *p* < 0.05; Figure 4), suggesting that aerobic exercise training for 12 weeks protected against obesity-induced apoptosis in the skeletal muscle.

## 4. Discussion

There were two major findings in the current study. First, we observed that exercise training attenuated obesity-induced skeletal muscle remodeling, indicating that the obesity-induced increase in the connective tissue of the skeletal muscle was reduced and that the decrease in the CSA was attenuated by exercise training. Second, we also found that exercise training in the obese mice protected against an obesity-associated increase in mitochondria-mediated apoptotic signaling (e.g., Bax/Bcl-2 ratio, mPTP opening sensitivity, and cytochrome c level) and apoptosis (cleaved caspase-3-positive cells and TUNEL-positive myonuclei) in their skeletal muscle. These data support the hypothesis that exercise training would attenuate obesity-related remodeling and apoptosis in obese skeletal muscles. Indeed, this is one of the few studies demonstrating that exercise training plays a protective role against long-term obesity-induced remodeling and apoptosis in the skeletal muscle.

We found that exercise training attenuated HFD-induced remodeling, such as decreased myocyte number and myofiber area (CSA), increased extramyocyte space, and IMCL infiltration in the skeletal muscle of the HFD + EX group (Figure 1 and Figure 2). According to previous studies, skeletal muscle remodeling occurs consistently with HFD, inducing diminished CSA [30], excessive extramyocyte space [31], and IMCL accumulation [32,33], which finally results in skeletal muscle dysfunction, including insulin resistance [34,35]. In the current study, skeletal muscle IMCL infiltration was rarely observed in the CON + EX in contrast to the CON group, under normal conditions, suggesting that the effects of exercise training on skeletal muscle IMCL infiltration were not evident in normal skeletal muscle. However, consistent with the findings of previous studies, we observed that the obese mice expressed greater degrees of skeletal muscle fibrosis and accumulation of IMCLs, which consequently lead to skeletal muscle dysfunction [36] and insulin resistance [29,37]. Furthermore, the current study demonstrated that exercise training for 12 weeks as a therapeutic intervention ameliorated obesity-induced skeletal muscle remodeling, including reduced CSA, increased extramyocyte space, and IMCL infiltration (Figure 1 and Figure 2). However, the 12-week exercise training did not attenuate the obesity-induced decrease in the number of myocytes (Figure 1B). The present data suggest that the number of myocytes cannot be recovered by exercise training because the skeletal muscles are post-mitotic tissues, although they undergo hypertrophy after obesity-induced atrophy.

We expected that exercise training would reduce mitochondria-mediated apoptotic signaling in the obese skeletal muscles. Given that the main cause of mitochondria-mediated apoptosis is oxidative stress or mPTP opening [38,39], we indirectly expected that mitochondria-mediated apoptosis could be induced by obesity as part of obesity-related remodeling, as previous studies have demonstrated that mitochondrial H_2_O_2_ production is enhanced and mPTP opening sensitivity is further increased by obesity [5]. Oxidative stress can accumulate pro-apoptotic proteins, such as Bax, resulting in increased mPTP opening sensitivity, apoptosome formation, and caspase-9- and caspase-3 activation, which finally leads to DNA fragmentation and programmed cell death [7,40]. Indeed, in the current study, the Bax/Bcl-2 ratio calculated using the Bax and Bcl-2 protein levels was markedly higher in the HFD-induced obese skeletal muscles than in the non-obese skeletal muscles (Figure 3C). This increased Bax/Bcl-2 ratio caused by obesity activated the mPTP opening (Figure 3D) and increased the cytochrome c (Figure 3E) and cleaved caspase-3 (Figure 4A) levels, consequently inducing DNA fragmentation, as previously demonstrated [18,41]. However, exercise training attenuated the obesity-induced apoptotic signaling, demonstrating that the Bax/Bcl-2 ratio, mPTP opening sensitivity, cytochrome c level, and cleaved caspase-3-positive cells in the obese skeletal muscles were reduced by exercise training.

Furthermore, we observed that exercise training ameliorated obesity-induced apoptosis, as indicated by TUNEL-positive myonuclei, which were clearly more abundant in the HFD-induced obese skeletal muscles, although exercise training did not affect the apoptosis markers (e.g., TUNEL-positive myonuclei) in the CON + EX group compared with the CON group (Figure 4B). However, we demonstrated that exercise training down-regulated apoptosis in the obese skeletal muscles (Figure 4B), suggesting that aerobic exercise, as a positive intervention, plays a therapeutic role in protecting against obesity-induced apoptosis in the skeletal muscle. These findings are inconsistent with the previous findings of Peterson and colleagues [20], who reported that apoptosis induced by obesity was attenuated by exercise training in the cardiac muscle but not in the skeletal muscle of a transgenic mouse model.

## 5. Conclusions

In conclusion, exercise training protected against obesity-induced remodeling in the skeletal muscles, including a decrease in the CSA and an increase in the connective tissue. In addition, although our study has a technical limitation in distinguishing between physiological apoptosis and pathological apoptosis, we found that aerobic exercise training for 12 weeks attenuated obesity-induced mitochondria-mediated apoptotic signaling (e.g., Bax, mPTP opening sensitivity, cytochrome c level, and cleaved caspase-3-positive cells) and apoptosis (e.g., TUNEL-positive myonuclei) in the skeletal muscle, suggesting that aerobic exercise training inhibits obesity-induced reduction of skeletal muscle mass. These findings support the hypothesis that modulation of mitochondria-mediated apoptosis by exercise training could attenuate obesity-induced skeletal muscle remodeling and thus reduce impairment of the skeletal muscle function with obesity.
